# *A. thaliana* Hybrids Develop Growth Abnormalities through Integration of Stress, Hormone and Growth Signaling

**DOI:** 10.1093/pcp/pcac056

**Published:** 2022-04-23

**Authors:** Katelyn Sageman-Furnas, Markus Nurmi, Meike Contag, Björn Plötner, Saleh Alseekh, Andrew Wiszniewski, Alisdair R Fernie, Lisa M Smith, Roosa A E Laitinen

**Affiliations:** Max Planck Institute of Molecular Plant Physiology, Am Mühlenberg 1, Potsdam-Golm 14476, Germany; Max Planck Institute of Molecular Plant Physiology, Am Mühlenberg 1, Potsdam-Golm 14476, Germany; Max Planck Institute of Molecular Plant Physiology, Am Mühlenberg 1, Potsdam-Golm 14476, Germany; Max Planck Institute of Molecular Plant Physiology, Am Mühlenberg 1, Potsdam-Golm 14476, Germany; Max Planck Institute of Molecular Plant Physiology, Am Mühlenberg 1, Potsdam-Golm 14476, Germany; Center of Plant Systems Biology and Biotechnology, Plovdiv 4000, Bulgaria; Max Planck Institute of Molecular Plant Physiology, Am Mühlenberg 1, Potsdam-Golm 14476, Germany; Max Planck Institute of Molecular Plant Physiology, Am Mühlenberg 1, Potsdam-Golm 14476, Germany; School of Biosciences and Institute for Sustainable Food, University of Sheffield, Western Bank, Sheffield S10 2TN, UK; Max Planck Institute of Molecular Plant Physiology, Am Mühlenberg 1, Potsdam-Golm 14476, Germany; Organismal and Evolutionary Research Programme, Faculty of Biological and Environmental Sciences, Viikki Plant Science Centre, University of Helsinki, Helsinki 00014, Finland

**Keywords:** *Arabidopsis thaliana*, Epistasis, Hybrid incompatibility, Natural variation, OAK, Receptor-like kinase

## Abstract

Hybrids between *Arabidopsis thaliana* accessions are important in revealing the consequences of epistatic interactions in plants. F_1_ hybrids between the *A. thaliana* accessions displaying either defense or developmental phenotypes have been revealing the roles of the underlying epistatic genes. The interaction of two naturally occurring alleles of the *OUTGROWTH*-*ASSOCIATED KINASE* (*OAK*) gene in Sha and Lag2-2, previously shown to cause a similar phenotype in a different allelic combination in *A. thaliana*, was required for the hybrid phenotype. Outgrowth formation in the hybrids was associated with reduced levels of salicylic acid, jasmonic acid and abscisic acid in petioles and the application of these hormones mitigated the formation of the outgrowths. Moreover, different abiotic stresses were found to mitigate the outgrowth phenotype. The involvement of stress and hormone signaling in outgrowth formation was supported by a global transcriptome analysis, which additionally revealed that *TCP1*, a transcription factor known to regulate leaf growth and symmetry, was downregulated in the outgrowth tissue. These results demonstrate that a combination of natural alleles of *OAK* regulates growth and development through the integration of hormone and stress signals and highlight the importance of natural variation as a resource to discover the function of gene variants that are not present in the most studied accessions of *A. thaliana*.

## Introduction

Reduced fitness in hybrids, due to epistatic interactions of parental genomes, is known as hybrid incompatibility. Hybrid incompatibilities have been reported in various plant species and the phenotypes range from embryo lethality to less severe phenotypes later in development resulting in reduced fertility. In the past decade, studies taking advantage of the genetic and molecular tools in *Arabidopsis thaliana* (Arabidopsis) have greatly enhanced our understanding of the mechanisms leading to different hybrid incompatibilities. While the most common type of hybrid incompatibility in Arabidopsis is hybrid necrosis (connected to an enhanced defense response), hybrids with embryo lethality, developmental irregularities such as altered shoot growth and development (Bikard et al. 2009, Smith et al. 2011, Alhajturki et al. 2018) and reduced photosynthesis (Plotner et al. 2017, Vaid et al. 2020) have also been reported. Understanding the genetic and molecular mechanisms resulting in different hybrid incompatibility phenotypes provides insights into the impact of genome interactions on plant adaptation and evolution.

Here, we report a new combination of parental genomes, which in the F_1_ hybrids results in non-parental shoot architecture and ectopic outgrowths on the leaf petioles. Shoot architecture is a combination of stem growth and lateral branch number and, by adjusting shoot habit, plants can adapt to limited light, different temperatures and nutrient availability. Shoot architecture is defined by the activity of the shoot apical meristem (SAM), the axillary meristems (AMs) and the vascular cambium (Ongaro and Leyser 2008, Stirnberg et al. 2010, Domagalska and Leyser 2011, Mueller and Leyser 2011, de Jong et al. 2014, Bennett et al. 2016, Shi et al. 2016). The SAM controls the vertical growth of the shoot, and the AMs support the growth of the lateral branches. The vascular cambium is responsible for the secondary thickening of the lateral growth. Plant hormones, mainly the interactions of auxin, strigolactones, cytokinins and gibberellic acid (GA; Teichmann and Muhr 2015, Wang et al. 2018), as well as sugars, are known to regulate the patterns of shoot branching (Barbier et al. 2015).

The hybrid incompatibility phenotype studied here is comparable to a reported hybrid incompatibility between Arabidopsis accessions Shahdara (Sha) and Blanes-1 (Bla-1). In Bla-1 x Sha F_1_ hybrids, an allelic interaction of a single *OUTGROWTH-ASSOCIATED KINASE* (*OAK*) gene caused aberrant outgrowths and altered shoot architecture (Smith et al. 2011). *OAK* is a member of the receptor-like kinase (RLK) gene family, with more than 600 genes in Arabidopsis encoding RLKs. The RLK proteins have a myriad of roles in mediating growth, development and stress responses in plants (Jose et al. 2020). *OAK* is located in a highly variable tandem array of closely related *RLK* genes (Smith et al. 2011), which contains four genes in the Col-0 reference genome. One of the members of the array has been duplicated to form tandem genes *At5g59670a* and *At5g59670b,* with the latter gene encoding OAK. Since *OAK* is not present in the reference genome (Col-0), it has not been characterized via loss-of-function mutant lines available for the reference background. However, *OAK* is present in one-third of Arabidopsis accessions.

*OAK* contains a tandem duplication of a malectin-like domain. Therefore, the protein has a similar structure to the *Catharanthus roseus* RLK1-like (*Cr*RLK1L, abbreviated to *Cr*RLK) family of proteins. There are 17 *Cr*RLK family proteins encoded in the Arabidopsis genome. Members of the *Cr*RLK family are involved in growth, development and reproduction and have roles in both responses to abiotic stresses and immunity (Galindo-Trigo et al. 2016, Jose et al. 2020). Recently, members of this family such as FERONIA (FER), ANXUR1 (ANX1), ANXUR2 (ANX2), THESEUS1 (THE1), HERCULES RECEPTOR KINASE1 (HERK1) and CURVY1 have been proposed to act as cell wall integrity sensors (Franck et al. 2018) and play a role in abiotic stress responses (Feng et al. 2018). For example, *FER*, one of the best-characterized members of this gene family, was recently shown to be involved in ABA and salt stress responses (Chen et al. 2016). In addition to the malectin-like domains, *OAK* contains a leucine-rich repeat (LRR) domain and therefore is also similar to the LRR-RLKs. Arabidopsis has 239 genes encoding LRR-type RLKs (Franck et al. 2018, Jose et al. 2020). LRR-RLKs contain tandem repeats of leucine residues that are involved in mediating growth, stress and immunity [reviewed in the work by Jose et al. (2020)]. LRR-RLKs are involved in abiotic stress response via the ABA pathway, such as RECEPTOR DEAD KINASE1 (Kumar et al. 2017) or via a role in cell-wall integrity sensing, such as LRR-RK MALE DISCOVERER 1-INTERACTING RECEPTOR-LIKE KINASE 2 (MIK2) (Van der Does et al. 2017). However, the function of OAK in development or stress signaling is not yet known.

Here, we present a detailed characterization of the F_1_ hybrid incompatibility case mediated by an allelic interaction of *OAK* between the Arabidopsis accessions Sha and Lag2-2. Our findings revealed that naturally occurring *OAK* alleles could regulate growth and development through the integration of specific hormones and stress signaling.

## Results

### OAK is necessary for the ectopic outgrowths observed in Sha × Lag2-2 F_1_ hybrids

**Fig. 1:**
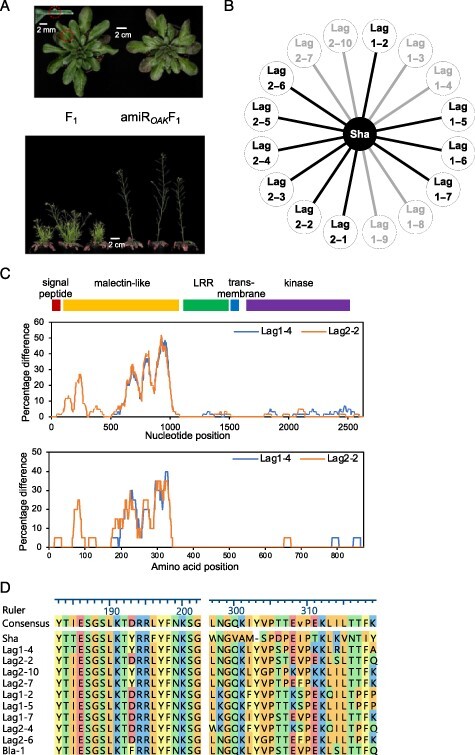
The ectopic outgrowths and altered shoot architecture in F_1_ hybrids between Sha and Lag2-2 are associated with OAK. (A) An example of ectopic outgrowth on the petiole adaxial side, plants at ∼28-leaf stage (red circle shows an outgrowth position on a leaf), and an example of an F_1_ rosette expressing an amiRNA targeting *At5g59670b* (amiR*_OAK_* F_1_). Silencing *OAK* also rescues the shoot phenotype (below). (B) The causal *OAK* allele is common in the Lagodechi area. Stronger lines represent a cross that results in the hybrid phenotype while progeny of crosses indicated with weaker line appear normal. Crosses were done in a reciprocal manner and hybrids were phenotyped in control conditions. (C) Divergence of *OAK* nucleotide identity and predicted amino acid similarity of Sha to Lag1-4 and Lag2-2, with sliding windows of 60 bp and 20 amino acids. (D) Alignment of OAK predicted protein sequences with amino acid change in non-synonymous SNPs for phenotype causing Lag accessions and Bla-1 (Blanes-1).

We identified that F_1_ reciprocal hybrids between the Arabidopsis accessions Shahdara (Sha) (Tajikistan) and Lagodechi 2-2 (Lag2-2) (Georgia) have a bushy dwarf phenotype and ectopic outgrowths on the leaf petioles. This aberrant growth phenotype is similar to that previously observed in F_1_ hybrids between Arabidopsis accessions Sha and Bla-1, which is caused by an allelic interaction of the *At5g59670b* gene encoding OUTGROWTH-ASSOCIATED KINASE (OAK) (Smith et al. 2011). The F_2_ segregation ratio of 1 : 1 of individuals with and without the outgrowth phenotype confirmed the linkage of the outgrowth phenotype to a single locus (**Supplementary Table S3**). Hence, we first tested if an allelic interaction of *OAK* was responsible for the phenotype in the Sha × Lag2-2 F_1_ hybrids. Using an artificial microRNA (amiR) approach (Schwab et al. 2006), we silenced *OAK* in the Sha parent (amiR*_OAK_* Sha), crossed to Lag2-2 and examined the resulting hybrids. If an interaction of the two *OAK* alleles was needed for the outgrowth formation, disrupting this interaction by silencing at least one of the alleles should diminish the outgrowth phenotype in the hybrids. The amiR*_OAK_* was designed to specifically target *OAK* and confirmed to reduce the expression of *OAK* in the hybrid (**Supplementary Fig. S1**). Indeed, progeny of amiR*_OAK_* Sha crossed to Lag2-2 (amiR*_OAK_* F_1_) did not have any outgrowths and were restored a typical shoot architecture (**Fig. 1A**). From this, we conclude that OAK is also necessary for the hybrid phenotype in Sha × Lag2-2 hybrids.

*OAK* is part of tandem gene duplication present in one-third of Arabidopsis accessions (Smith et al. 2011). To investigate the *OAK* incompatibility in more detail, we performed crosses between the Sha accession and 16 additional accessions collected from Lagodechi, Georgia, that were available in the Arabidopsis seed bank (Nottingham Arabidopsis Stock Centre; NASC). From these crosses, 10 hybrids showed the incompatible phenotype with outgrowths and aberrant shoot habit, whereas six resembled the parents (**Fig. 1B**). This indicates that functionally different *OAK* alleles are present in the Lag population. Comparison of the Sha, Lag1-4 and Lag2-2 genomic *OAK* sequences revealed that the only non-synonymous polymorphisms encoding non-conserved residues unique to Lag2-2 were observed in the region encoding the malectin-like domain of the gene (**Fig. 1C**). Similarly, the malectin-like domain was previously indicated to be hypervariable between the Sha and Bla-1 alleles, which are also known to give the outgrowth phenotype (Smith et al. 2011). In the Sha x Bla-1 study, the region containing the malectin-like domain was found to be under positive selection suggesting an adaptive role in nature. To further investigate if the polymorphisms in the malectin-like domain were associated with the outgrowth phenotype in the Lag population, we sequenced a 1 kb region encompassing the domain in selected Lag-individuals known to either induce an outgrowth phenotype when crossed to Sha or not. Only two Single nucleotide polymorphisms (SNPs) in this region showed a correlation with the outgrowth phenotype (**Fig. 1D**). At position 577 of the coding sequence, a change of T to G causing an amino acid change from tyrosine to aspartic acid (Y193D) was observed (or a T to X nucleotide substitution causing a Y193F change in Bla-1), along with a change of C to T at position 916 causing an amino acid change from proline to threonine (P306T). We therefore speculate that these two amino acid changes may contribute to the hybrid incompatibility.

### Altered growth and vascular organization in F_1_ hybrids

**Fig. 2:**
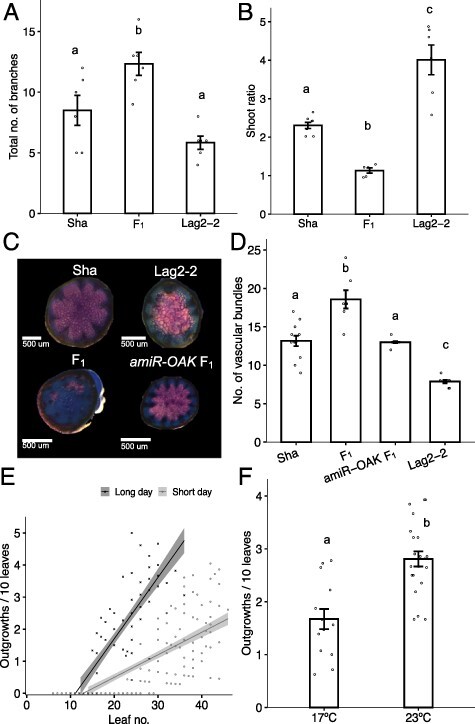
Detailed characterization of the Sha × Lag2-2 F_1_ shoots. (A) Total average number of basal and lateral branches of parents and hybrid. (B) The ratio of main stem height (centimeters) to lateral and basal branch height (centimeters). Plants were grown at 21°C, LD conditions following a 4-week vernalization period for Lag2-2. For (A) and (B), *N* = 5, 6 and 6 plants. Letters represent Tukey’s honestly significant difference (HSD) adjusted *P*-values < 0.05 from a one-way ANOVA. (C) Visualization of vascular structure with 0.1% toluidine blue. (D) Number of vascular bundles in mature stems, counted from sections stained with 0.1% toluidine blue. *N* = 12, 7, 8 and 13. Letters represent Tukey’s HSD adjusted *P*-values < 0.05 from a one-way ANOVA. (E) Outgrowth appearance in LD and SD conditions. *N* = 13 and 14. Lines represent linear regression with 95% confidence shaded bands. (F) Hybrid plants grown at 17°C and 23°C constant conditions. *N* = 14 and 22. Letters represent a *P*-value < 0.001 from a two-tailed *t*-test. In all subfigures, bars represent the standard error of the mean (SEM).

To understand the functional role of OAK in the hybrids, we first examined the shoot phenotype of the hybrids and parents more in detail. The hybrids had an average of 57% more branches than the parents (**Fig. 2A**). Furthermore, the lateral and basal branches of the F_1_ hybrids grew taller than the main shoot resembling a loss of apical dominance phenotype, as quantified by the shoot : height ratio (**Fig. 2B**).

Next, to find out if altered shoot growth is associated with altered shoot vasculature, we analyzed the cellular patterning of the stems. Transverse sections of the hybrid and parents’ stems, stained with toluidine blue to highlight lignin, revealed that the cellular patterning of the hybrid stem was disorganized with vascular bundles and lignification occasionally occurring in the pith (**Fig. 2C**, **Supplementary Fig. S2**). Moreover, the increased number of vascular bundles in hybrids compared to the parents (**Fig. 2D**) suggests altered vascular development in the hybrids. Furthermore, the vascular bundles of the amiR*_OAK_* Sha × Lag2-2 resemble the Sha parent, indicating that OAK is required for the change in vascular patterning in Sha × Lag2-2 hybrids.

Secondly, we inspected the ectopic outgrowths. In long-day (LD) conditions (16 h/8 h; 21°C/17°C, which unless otherwise stated are referred to as control conditions), the first outgrowth appeared after the production of 16 leaves. To investigate if the initiation of outgrowth formation was connected to the transition to flowering, we grew the hybrids and the parents under short-day (SD) (8 h/16 h) and lower temperature (17°C constant) conditions, which both are known to delay flowering time. In both conditions, the first outgrowth appeared later than in the control condition. The first outgrowth appeared in SD conditions at 28 ± 3 leaves and at 22 ± 1 leaves in 17°C conditions (**Fig. 2E**, **Supplementary Table S5**). In addition, the hybrids grown in SD conditions or at a lower temperature had a significantly reduced total number of outgrowths than the plants grown in LD conditions (**Fig. 2E**, **F**). These results suggest that the initiation of outgrowth formation is associated with the developmental switch from vegetative to reproductive growth while the total number of outgrowths depends on the growth rate. Altogether, we conclude that the OAK-mediated hybrid incompatibility phenotype in the Sha × Lag2-2 F_1_ hybrids is controlled by both environmental and developmental cues.

### Stress mitigates the outgrowth formation in hybrids

**Fig. 3:**
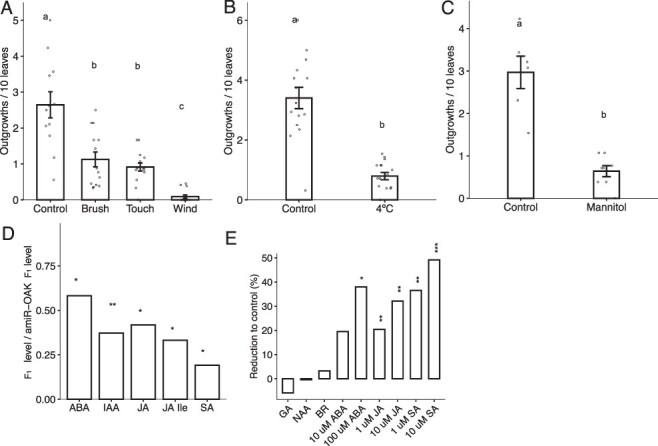
The role of stress and hormone signaling in the outgrowth phenotype. (A) The number of outgrowths in F_1_ hybrid plants when grown in control greenhouse conditions and touched with a gloved hand or brushed with a 2-cm brush 10 times per day or had 2.3 m/s wind applied to them with a fan. Letters represent Tukey’s (HSD) adjusted *P*-values < 0.05 from a one-way ANOVA, *N* = 14, 12, 15 and 14. (B) Outgrowth number in plants that were grown at 21°C after 4 weeks of 4°C treatment, compared to plants grown at 21°C under our control conditions without cold treatment. *N* = 15, 16. (C) The effect of mannitol stress on outgrowth number. F_1_ hybrids grown in pots were subjected to watering with 125 mM mannitol once per day for two weeks after the 4-leaf stage and compared to plants watered without mannitol. N = 7 and 8. Letters from B and C represent a *P*-value < 0.001 from a two-tailed *t*-test. (D) Abundance of hormones measured in petiole and outgrowth tissue, shown as a ratio of F_1_ hybrids to amiR*_OAK_* F_1_. *N *= 4. (E) Reduction of the number of outgrowths in the hybrids that were sprayed with GA, NAA, BR, ABA, JA and SA for 2 weeks. *N* > 16 for each treatment. In D and E, asterisks represent *P*-values of < 0.05 (*), < 0.01 (**) and < 0.001 (***) as calculated from a one-tailed *t*-test of each treatment against the control group. In all subfigures, bars represent SEM.

Receptor-like kinases typically mediate both growth and stress responses (Jose et al. 2020). Therefore, we asked if the outgrowth formation of the F_1_ hybrid is affected by stress. First, we observed that none of the common pathogen markers were expressed in the hybrids indicating that the phenotype is not due to induced defense responses (**Supplementary Fig. S4**). Then, we subjected the F_1_ hybrids and parents to three mechanical stresses: touch, brush and wind. For touching and brushing, the leaves were patted with a gloved hand or brushed with a 2-cm paintbrush 10 times daily for 2 weeks starting from when they had 6–8 leaves. For wind stress, plants were exposed to a 2.3 m/s wind generated by a fan and applied horizontally to the plants for 16 hours per day. To minimize technical variation, all experiments included at least 14 replicates. The number of outgrowths was scored every other day. We found that all three mechanical stresses reduced the total number of outgrowths by at least 50% (**Fig. 3A**). The wind treatment had the most potent effect, with no outgrowths on 80% of the plants and the remaining 20% of plants only produced a single outgrowth.

We then asked if OAK affects outgrowth formation by sensing and signaling of the touch stress or whether the touch stress signaling affects outgrowth formation downstream of OAK. We reasoned that if OAK was upstream of the touch stress signaling, induction of the expression of the known touch sensing genes would be abolished in the ami*_OAK_*F_1_ line in which *OAK* is silenced. However, if touch sensing and signaling were upstream of OAK, then touch stress would be induced in both F_1_ and ami*_OAK_*F_1_. To investigate these scenarios, we measured transcript levels of 6 known touch-responsive genes in F_1_ and in amiR*_OAK_* F_1_ plants that had been touched with a gloved palm 10 times for three days. Our results showed that several of the known touch responsive genes were induced in both lines upon touching in comparison to non-touched control plants (**Supplementary Fig. S3**) suggesting that OAK acts downstream in touch response signaling events.

Next, we tested the role of other abiotic stresses on the outgrowth formation. We exposed the hybrids to a cold non-freezing temperature (+4°C) for 4 weeks, whereafter they were grown at the control conditions (LD, 23°C). The cold-exposed hybrids had 76% fewer outgrowths than those grown only in the control conditions (**Fig. 3B**). Third, we tested the effect of osmotic stress on the outgrowths. We watered the hybrids with 125 mM mannitol for 2 weeks. Mannitol-treated plants had 80% fewer outgrowths in comparison to the untreated control plants (**Fig. 3C**). Altogether, these results indicate that reduction in *OAK*-induced outgrowth formation in the hybrids is a general stress response rather than a specific response to a particular type of stress.

### Outgrowths are associated with reduced levels of hormones

To investigate the role of hormone signaling in the outgrowth formation, we quantified the endogenous levels of indole-3-acetic acid (IAA; auxin), abscisic acid (ABA), salicylic acid (SA) and jasmonic acid (JA) in F_1_ petioles with outgrowths and in *amiR_OAK_* F_1_ petioles without outgrowths. We found that the levels of all measured plant hormones (IAA, ABA, SA and JA) were significantly reduced in the outgrowth tissue compared to the control tissue (**Fig. 3D**).

We reasoned that if reduced hormone levels in hybrids are required for the outgrowth phenotype, the exogenous application of these hormones should reduce the number of outgrowths. In addition to auxin (1-naphthalene acetic acid NAA), ABA, SA and JA, we included GA and epibrassinolide in our experiment. We sprayed the F_1_ plants for 2 weeks starting at the 4-leaf stage and counted the number of outgrowths per 10 leaves. Control plants were sprayed with an equivalent amount of Tween-20 and ethanol carrier. Application of NAA (1 mM), GA (100 µM) and BR (1 µM) did not affect the number of outgrowths (**Fig. 3E**), whereas the application of SA (1 and 10 µM), JA (1 and 10 µM) and ABA (100 µM) mitigated the outgrowth phenotype. The number of outgrowths was reduced by ∼50% when sprayed with 10 µM SA and by ∼35% when sprayed with 10 µM JA and 100 µM ABA in comparison to the control-treated hybrids (**Fig. 3E**). A dosage response effect was noted for ABA, JA and SA (**Fig. 3E**). These data support that the reduction of hormones in hybrid petioles underlies the outgrowth phenotype, further suggesting that the OAK regulates development and growth via hormone signaling.

**Fig. 4:**
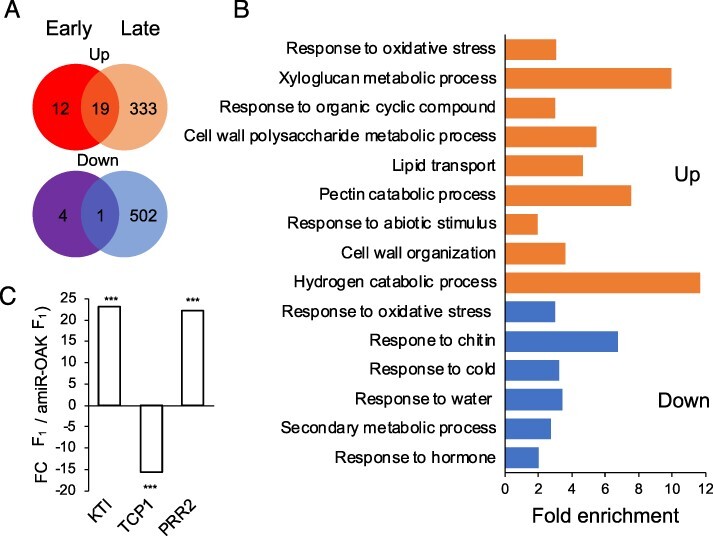
Transcriptome analysis of F_1_ and amiR*_OAK_* F_1_ leaf petioles. (A) Venn diagram of DEGs in the F_1_ hybrid samples in comparison to the amiR*_OAK_* F_1_ line at the two time-points (log_2_ Fold Change > 1, *P*_adj_ < 0.05). (B) GO enrichment of all DEGs. Fold enrichment calculated using the background reference dataset in the AgrigoV2 database, significance level < 0.01 Fischer’s exact test. (C) Validation of selected genes from the mRNA sequencing experiment. Fold change of selected genes (F_1_ to amiR*_OAK_* F_1_). Fold change calculated from four biological replicates with two technical replicates, *** represents a *P*-value of < 0.001 from a two-tailed *t*-test.

### mRNA-seq reveals transcriptional changes associated with the outgrowth phenotype

**Fig. 5:**
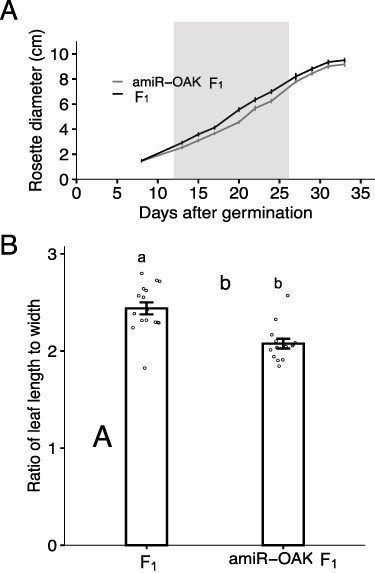
F_1_ hybrids are impaired in growth. (A) Rosette growth profile of F_1_ plants and amiR*_OAK_* F_1_. *N* = 19 and 20, bars represent SEM. Shaded area represents significance with a *P*-value of < 0.01 between F_1_ plants and amiR*_OAK_* F_1_. (B) Leaf shape of F_1_ hybrids and amiR*_OAK_* F_1_. Ratio represents the length : width measurement of the 5th rosette leaf *N* = 16 and 14. Letters represent a *P*-value < 0.001 from a two-tailed *t-*test, bars represent SEM.

To gain further insights into the role of OAK in the outgrowth phenotype, we conducted an mRNA-sequencing (mRNA-seq) analysis comparing pooled petiole tissue of F_1_ hybrids containing outgrowths and pooled petioles from the amiR*_OAK_* F_1_ with no outgrowths. Using the amiR*_OAK_* F_1_ line in the comparison, which has reduced *OAK* transcript levels, allows us to control for effects of hybridization that are not associated with the OAK-caused phenotype. We sampled at two different time points; firstly, when the rosettes had 22–24 leaves (early), and secondly when the plants had 26–28 leaves (late). At the early time point, the outgrowths were starting to form but visible enough to allow reliable excision of outgrowth tissue. It was selected to capture the gene expression changes that potentially reflect to both changes causal for outgrowth formation and changes that are a result of outgrowth formation. At the late time point, it is expected that most gene expression changes will reflect the process of outgrowth formation rather than causal changes that initiate outgrowth formation. Differentially expressed genes (DEGs) were identified by comparing mRNA transcript levels in the petiole tissue of Sha × Lag2-2 F_1_ plants with outgrowths to the control amiR*_OAK_* F_1_ plant independently at both time points (Log_2_ Fold Change > 1.0, adjusted *P*_adj_  < 0.05). In the early outgrowth tissue, 30 genes were upregulated and 5 downregulated compared to the early control tissue. 351 DEGs were upregulated and 503 were downregulated in the late outgrowth tissue compared to the late control tissue (**Fig. 5A**, full DEG list **Supplementary Table S6**).

To gain a better insight into which pathways OAK may be involved in, we did a gene ontology (GO) analysis for the 363 upregulated and 507 downregulated DEGs in either the early or late time point. Among the upregulated DEGs, we found an enrichment of 32 GO terms with key child terms being: response to abiotic stress, response to oxidative stress, organic cyclic compound metabolism, lipid transport, cell wall organization, hydrogen peroxide catabolic process and xyloglucan metabolic process (**Fig. 5B**, Agrigo V2, enrichment value < 0.01, complete list of GO terms in **Supplementary Table S4**). This suggests that altered cell wall growth could play a role in the outgrowth formation. In the downregulated transcripts, 22 GO terms, including response to chitin, response to hormones, secondary metabolic processes, response to oxidative stress, response to cold and response to water deprivation were found. Chitin is associated with defense responses against fungi (Antolin-Llovera et al. 2012, Monaghan and Zipfel 2012) but chitin signaling has also been linked to abiotic stresses, particularly to salt stress (Espinoza et al. 2017).

Our analysis identified 20 genes that were differentially expressed in both early and late outgrowth samples in comparison to their controls (**Supplementary Table S7**). Of these, 19 were upregulated and only one was downregulated. Six of the 19 upregulated genes encode lipid transfer family proteins of unknown function. Other genes that were upregulated in both the early and late outgrowth timepoints are *AT5G64110* (*PEROXIDASE 70), AT5G58390* (unknown peroxidase family gene) and *AT5G57240* (*OXYSTEROL BINDING PROTEIN-RELATED PROTEIN 4C, ORP4C*), *AT1G36060* (*TRANSLUCENT GREEN3, TG/WOUND INDUCED DEDIFFERENTIATION 3, WIND3*), *AT1G73260* (*KUNITZ INHIBITOR1, KTI1)* and *AT4G02700* (*SULFATE TRANSPORTER 3.2*). *ORP4C* and *TG/WIND3* are associated with abiotic stress response in plants (Huang et al. 2008, Zhu et al. 2014), with *ORP4C* also being associated with JA and SA signaling (Brodersen et al. 2006) and *KTI1* has been implicated in defense responses (Arnaiz et al. 2018).

In both early and late outgrowths, the only downregulated gene was *AT1G67260*, encoding a TCP transcription factor family protein TCP1. The downregulation of *TCP1* in the outgrowths was confirmed using Real-time quantitative PCR (RT-qPCR) along with two genes upregulated at both time points *AT1G73260* (*KTI1*) and *AT4G13660* (*PRR2*) (**Fig. 4C**). *TCP1* was first identified in the regulation of symmetric development of flower organs, but since then, it has been shown to regulate the longitudinal growth of leaves (Cubas et al. 2001, Busch and Zachgo 2007, Wang et al. 2020, Klionsky et al. 2021). In addition, it is involved in BR biosynthesis (Guo et al. 2010) and in strigolactone signaling (Wang et al. 2020).

Due to the role of *TCP1* in regulating leaf growth and shape, we wanted to examine if the reduced expression of *OAK* in the amiR*_OAK_* F_1_ line influenced leaf growth. First, we compared the early rosette diameter of F_1_ hybrids and amiR*_OAK_* F_1_ hybrid plants grown under control conditions. The hybrids had an increased rosette diameter beginning at 12 d of growth in comparison to the amiR*_OAK_* F_1_ plants (**Fig. 5A**). However, final rosette size was comparable in both lines. Second, we looked at the leaf shape by measuring the ratio between the length and the width of the fifth leaf of the F_1_ hybrid and amiR*_OAK_* F_1_. The leaf length : width ratio was significantly higher in the F_1_ hybrids indicating that the F_1_ hybrid leaves are narrower than in the amiR*_OAK_* F_1_ plants (**Fig. 5B**). Taken together, the allelic interaction of *OAK* in the F_1_ hybrid leads to altered leaf growth. Whether this is due to reduced expression of *TCP1* in the F_1_ hybrids requires further investigations.

## Discussion

We have shown that the allelic interaction of the *OAK* gene is required for altered shoot development in the F_1_ hybrid of the Sha and Lag2-2 accessions of Arabidopsis, with a reduced height of the main stem, increased branching and outgrowth formation on the leaf petioles. Furthermore, reduced levels of plant hormones IAA, ABA, SA and JA in the leaf petioles underlie the outgrowth phenotype indicating that the hybrid phenotype is likely regulated by a complex interaction between plant hormone pathways. ABA is considered a positive regulator of abiotic stress but was also more recently demonstrated to have roles in non-stress mediated growth responses (Yoshida et al. 2019). SA and JA, in addition to their roles in abiotic stresses (Scott et al. 2004, Hu et al. 2013), are well-known positive regulators of defense responses (Yang et al. 2019). In addition to acting on their own, these three hormones are known to have complex, context-specific interactions (Aerts et al. 2021). However, there does not appear to be an overactivation of the defense response. Neither the mRNA sequencing data nor the RT-qPCR analysis (**Supplementary Fig. S4**) showed an induction of defense-related genes. Moreover, we found that abiotic stress (including mechanical, temperature and osmotic stresses) diminished the effects of OAK. Therefore, the most likely explanation is that OAK through downregulation of SA, JA and ABA leads to an altered stress response and abnormal growth. This was further supported by the reduced number of outgrowths in the hormone-treated hybrids. Taken together, our results suggest that OAK plays a role in the integration of growth and development through altered hormone signaling in the hybrid rather than direct involvement of OAK in stress signaling.

Our mRNA sequencing analysis revealed 20 genes that were differentially expressed in both the early and late outgrowths. While most of the genes were found to be related to abiotic stress and/or hormone signaling, *TRANSLUCENT GREEN3/WOUND INDUCED DEDIFFERENTIATION 3* (*TG/WIND3*) and its homologs have been implicated in induction of callus formation (Iwase et al. 2011), possibly suggesting that callus formation could be a reason for outgrowth formation. However, previous findings by Smith et al. (2011) have stated that the outgrowths did not constitute undifferentiated callus. In addition, the analysis of transverse sections of the hybrid petioles with outgrowths and the parent petioles without outgrowths did not show a statistically significant difference in the cell number between the hybrid and the parent indicating that the outgrowth tissue originates from elongating parenchyma tissue and not an increase in the number of cells (**Supplementary Fig. S5**). Altogether these results do not indicate activated callus formation in the outgrowths.

*TCP1* was the only gene that was downregulated in both early and late outgrowth tissues. TCP1 was initially identified to regulate symmetric development of flower organs, but since then it has been shown to also regulate the longitudinal growth of leaves (Cubas et al. 2001, Busch and Zachgo 2007, Koyama et al. 2010, Wang et al. 2020, Zheng et al. 2021). Indeed, we observed that the hybrids had a larger early rosette diameter and altered leaf shape (**Fig. 5**). TCP1 is known to positively regulate DWF4, a transcription factor involved in BR signaling and SMAX-LIKE1 proteins involved in strigolactone signaling (Guo et al. 2010, Wang et al. 2020, Zheng et al. 2021). However, we did not observe any change in transcripts related to BR or strigolactone signaling indicating the reduced expression of *TCP1* in outgrowth tissue is likely mediated by other signals (**Supplementary Fig. S4**). Taken together, these results indicate that OAK, through TCP1, could play a role in leaf development under normal growth conditions. Destabilization of TCP1, induced by phytoplasma infestation, is also associated with altered shoot architecture (Chang et al. 2018) providing a possible link between the leaf and shoot phenotypes. The specific role of TCP1 in the hybrid phenotype will require further investigation.

The OAK protein has both LRR and malectin-like domains, displaying similarity to the very large and functionally diverse LRR kinase family as well as the *Cr*RLK1L family of proteins. Analysis of *OAK* sequences in nine Lag accessions originating from the same area revealed that there are functionally different alleles of *OAK* present in the Lag population. Furthermore, we pinpointed two non-synonymous SNPs in the malectin-like domain, which correlate with whether a cross of the accession to Sha results the abnormal phenotype. The *Cr*RLK1L kinases often have roles in cell wall integrity, cell expansion and stress responses. The most well-characterized example is FER, which is known to integrate several cell wall, stress and hormone pathways (Liao et al. 2017). Another example of the *Cr*RLK1L family is THE1, which interacts with the JA/SA and lignification pathways during defense responses (Qu et al. 2017). Most *Cr*RLK1L proteins interact with small peptides or cell wall epitopes (Feng et al. 2018, Galindo-Trigo et al. 2020). It will be interesting to investigate whether the SNPs associated with the outgrowth phenotype in the Lag_*OAK*_ gene region encoding the malectin-like domain could be involved in functions similar to FER or THE1 such as binding extracellular small peptides or cell wall components. These SNPs may also affect Lag_*OAK*_ interactions with other members of the LRR-RK family as they are known to form homo- and hetero-dimers (Wang et al. 2016, Xi et al. 2019).

To conclude, our results demonstrate that a combination of natural alleles of *OAK* regulates growth and development through the integration of hormone and stress signals. Significantly, our findings highlight the importance of natural variation as a source to discover the function of non-reference alleles and explore their interactions in plant adaptation and their potential role in evolution.

## Materials and Methods

### Plant lines and growth conditions

Seeds from all Arabidopsis accessions used in this study are listed in **Supplementary Table S1**, are publicly available and were ordered from NASC. Seeds were stratified in 0.1% (w/v) agarose in water at 4°C in the dark and then sown onto soil. Plants were grown either in LD conditions (16 h/8 h; 23°C/ 17°C) or SD conditions (8 h/16 h; 23°C/ 17°C). For testing the effect of temperature, plants were grown at constant temperatures of 17°C and 23°C under LD conditions. In all conditions, light intensity was 150 µE m^−2^ s^−1^ and humidity 60–70%. Unless otherwise noted, outgrowths were scored when the plants had 28 leaves and the number of outgrowths per 10 leaves was counted. The shoot phenotypes were scored after siliques began to form. To generate F_1_ hybrids, Lag2-2 was used as the pollen donor, with the exception that reciprocal crosses were performed to check for hybrid phenotypes of Sha with other accessions of the Lagodechi region.

### Generation and selection of transgenic plants and hybrids

Artificial microRNA lines silencing *OAK* are as described in (Smith et al. 2011). For initial phenotyping, three independent transformants were generated in the Sha accession via *Agrobacterium tumefaciens* (GV3101) using the floral dip method (Clough and Bent 1998),

T_0_ seeds were selected on BASTA plates (10 mg/L) and the resistant plants were confirmed for the presence of the transgene (using primers specific for pFK210 and the artificial microRNA; primers are listed in **Supplementary Table S2**). The three independent transformants were crossed to Lag2-2 and the resulting hybrids were phenotyped. The hybrids were confirmed using SSLP markers NGA225. DNA was isolated using the Cetyltrimethylammonium Bromide method as described in (Doyle and Doyle 1987).

### Transcriptome sequencing and analysis

For the transcriptome analysis, we took advantage of the segregation of the amiRNA in the F_1_ progeny of crosses between Lag2-2 and the amiRNA*_OAK_*Sha line. The F_1_ hybrid progeny segregate between those with silenced *OAK* that do not show any outgrowths and those without the transgene that form outgrowths. The heterozygosity of the cross and the presence or absence of the transgene was confirmed by genotyping as described above. Plants not containing the transgene were harvested for petioles with outgrowths. Petioles from plants with the transgene that did not form the outgrowths were used as controls. At the 22–24 leaf stage, early outgrowths were collected, and similar petiole tissue was harvested from the control plants and frozen in liquid nitrogen. At the 26–28 leaf stage, outgrowths were harvested along with control tissue and considered as late outgrowths. RNA was isolated with the QIAGEN RNAeasy Plant Mini Kit with on-column DNase treatment prior to sequencing. mRNA sequencing was carried out by BGI Genomics (www.bgi.com). Low-quality reads and reads with uncalled bases (unknown bases greater than 5%) were discarded. Clean reads were assembled and mapped using hierarchical indexing for spliced alignment of transcripts (HISAT) (Kim et al. 2015) and fast gapped-read alignment with Bowtie (Langmead and Salzberg 2012). The gene expression analysis was calculated using RNA-seq by expectation-maximization (RSEM) (Li and Dewey 2011), and DEGs were detected using DEseaq2 based on a negative binomial distribution (Love et al. 2014). Parameters were set to fold change > 2, adjusted *P*-value < 0.05.

### GO enrichment

GO enrichment analysis was performed using Agrigo V2 (http://systemsbiology.cau.edu.cn/agriGOv2/) plant Singular Enrichment Analysis program with the complete GO database. DEGs that were up or downregulated were treated separately, with a significance level of 0.01 for Fischer’s exact test, and with the Benjamini–Yekutieli multi-test adjustment used for FDR. A full list of all GO terms is in **Supplementary Table S4**.

### RT-qPCR

RNA was extracted from pools of two plants each, unless otherwise indicated, using the Qiagen Plant RNeasy Mini kit. One microgram of RNA was DNase-treated with the same on-column kit as used for the mRNA-seq analysis. Complementary DNA was synthesized with oligoDT primers (ImProm II kit) according to the manufacturer’s instructions. Real-time quantitative PCR (RT-qPCR) was performed using SYBR green PCR Mastermix and an AbiPrism 7900HT machine. At least three biological replicates were used with two technical replicates for each condition. Expression was normalized to two housekeeping genes, *SAND* and *GAPDH*. Primers are listed in **Supplementary Table S2**.

### Hormone extractions, measurements and treatments

Petioles of F_1_ hybrids and amiR*_OAK_* F_1_ hybrids were harvested from plants at the 26–28 leaf stage at midday. Four replicates each with pools of two plants per replicate were extracted and endogenous levels of IAA, SA, JA, JA-lle and ABA were measured as in Salem et al. (2016, 2020). For foliar spray experiments, plants at the 4–6 leaf stage were sprayed with the described concentration of hormone and 0.02% Tween-20. Control plants were sprayed with water with 0.01% ethanol (as the hormone stock solutions were prepared in ethanol) and 0.02% Tween-20 added. Two weeks later, plants were scored 3 d a week for outgrowth and leaf number. Final rosette diameter was measured after 2 weeks. NAA (Duchefa Biochemie), SA (S-7401, Sigma-Aldrich), JA (M1068-5G, TCI Deutschland), eBR (E1641, Sigma-Aldrich), GA (Duchefa Biochemie) and ABA (Sigma-Aldrich) were used.

### Vascular bundle measurements

To analyze vascular bundle organization, the main floral stems from mature plants were cut within 2 cm of the base with a razor blade and sectioned by hand. Sections were stained with 0.1% (w/v) toluidine blue solution and visualized with a Leica M165 FC microscope using brightfield imaging. The number of vascular bundles was counted from the images of the stained sections.

## Supplementary Material

pcac056_Supp

## Data Availability

The data generated or analyzed during the current study are available from the corresponding author on reasonable request. The RNAseq data have been submitted to the NCBI SRA database with reference ID PRJNA771496 (link https://www.ncbi.nlm.nih.gov/bioproject/771496).
