# Supplementary material for: A. thaliana Hybrids Develop Growth Abnormalities through Integration of Stress, Hormone and Growth Signaling
Source: Plant Cell Physiol. 2022 Apr 23;63(7):944–54. doi: 10.1093/pcp/pcac056 (PMC9282726; doi:10.1093/pcp/pcac056)
Supplement: pcac056_Supp [file pcac056_supp.zip › pcp-2022-e-00004-File007.pdf]

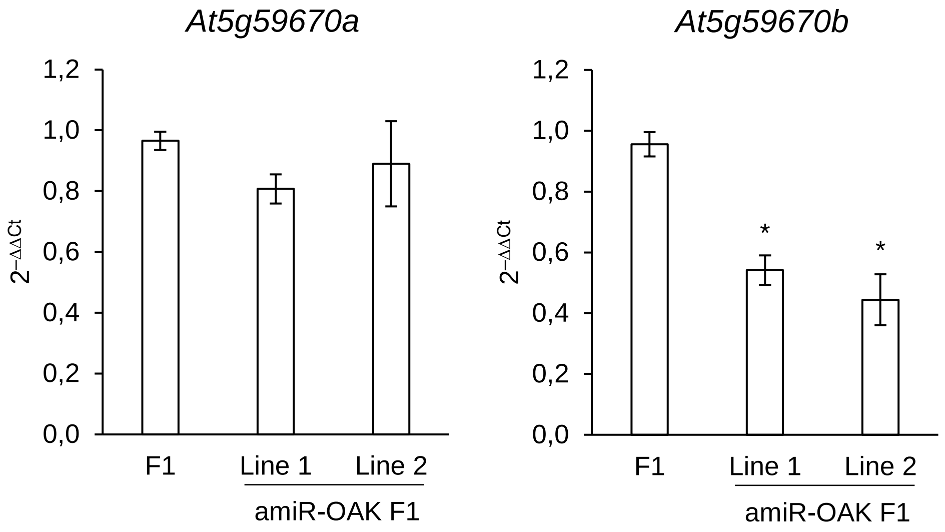

Figure S1. RT-qPCR analysis of *OAK* in the F_1 hybrid and two independent amiR$_{OAK}$ F_1 lines.

Expression level of *At5g59670a* and *At5g59670b* in the petiole tissue were measured relative to *UBC* (*At5g25760*) reference gene. $2^{-\Delta\Delta Ct}$ is representative of three biological replicates of two plants each, with two technical replicates. Asterisks represent p-values < 0.01 (*), bars represent SE.

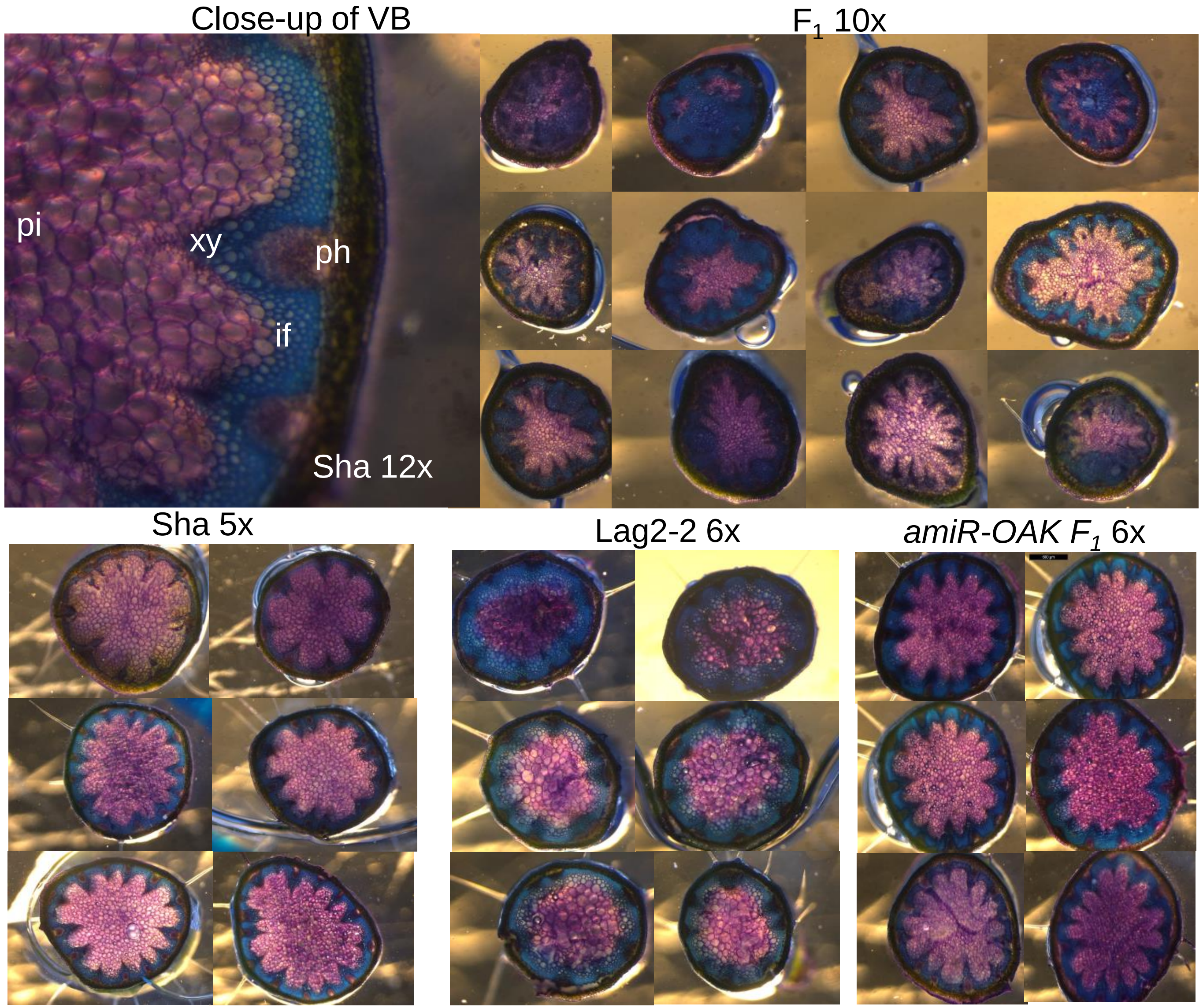

Figure S2. Example images of transverse sections of mature Sha, Lag2-2, F_1 and amiR$_{OAK}$ F_1 hybrid stems. Mature plants were cut within 2-cm of the base of the stem and sectioned by hand. For visualization with light microscopy, the sections were stained using toluidine blue. The number of vascular bundles were counted by hand and the magnification that was used for visualization is indicated in the figure. Each section represents one plant. Abbreviations are as follows: pi = pith, xy = xylem, ph = phloem, if = interfascicular fibers.

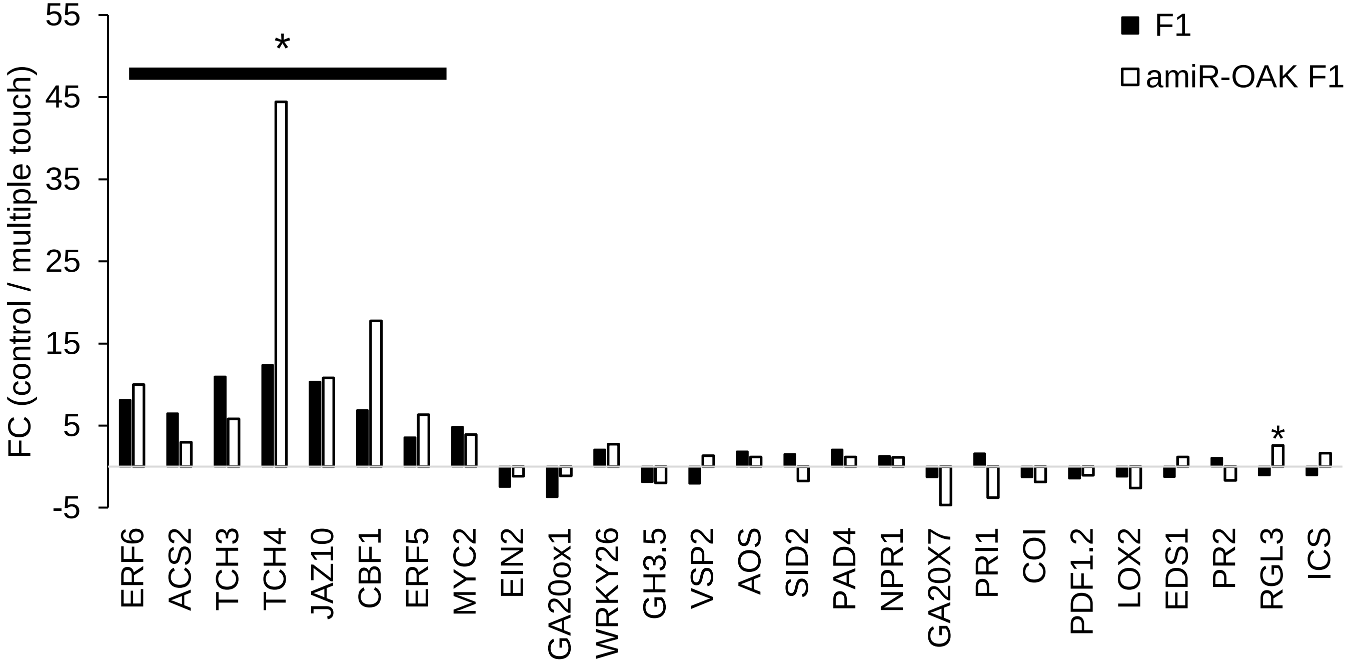

Figure S3. RT-qPCR analysis of touch-related genes in leaf petioles of F_1 hybrids and amiRNA$_{OAK}$ F_1 hybrids. The tissue of leaf petioles of F_1 hybrids and amiRNA$_{OAK}$$F_1$ hybrids were sampled at 30 minutes after the third day of touching 10 times daily with a gloved hand. FC is representative of three biological replicates of two plants each, with two technical replicates. FC is shown between the touched plants and control plants that were not touched. Asterisk represents genes that are significantly induced by touch, two-tailed t-test p-value < 0.05.

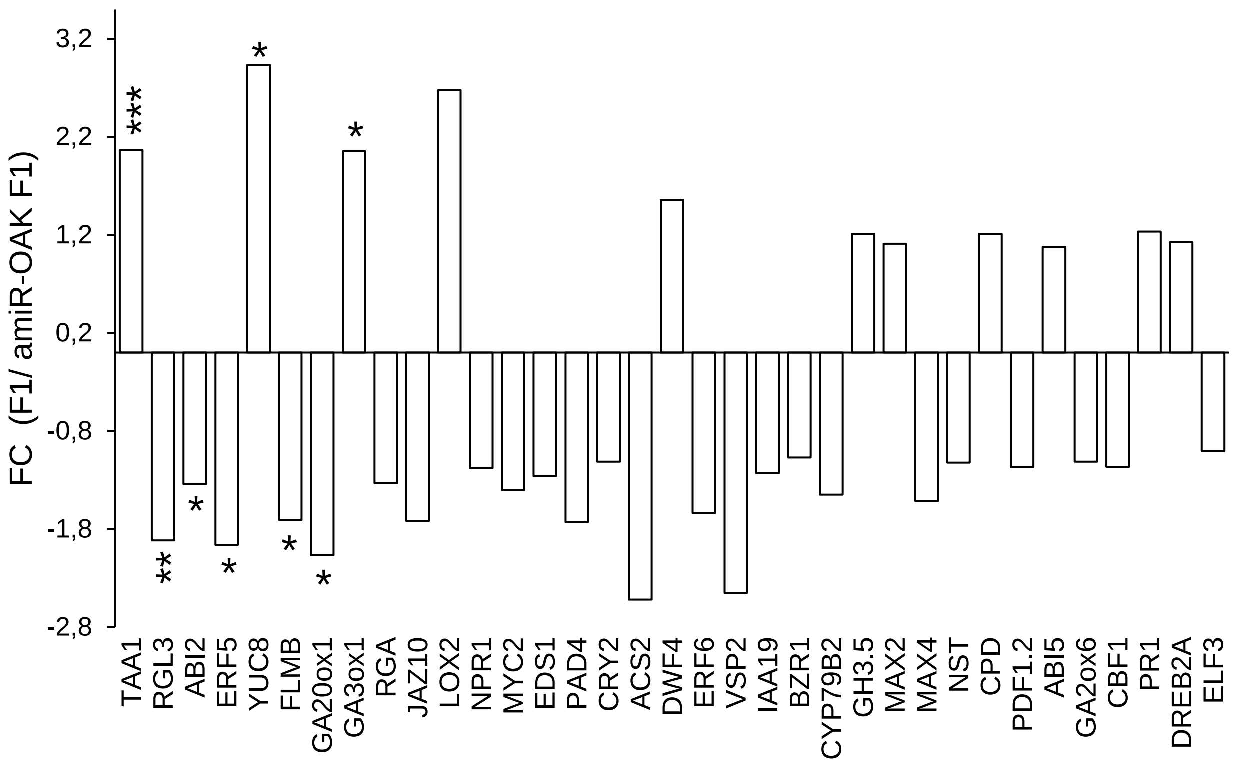

Figure S4. RT-qPCR analysis of select hormone- and defence-related genes. Fold change (FC) of 35 genes between F_1 and amiRNA$_{OAK}$ F_1 hybrids are shown. FC is representative of four biological replicates of two plants each, with two technical replicates of petiole tissue. Asterisks represent p-values < 0.05 (*), 0.01 (**), 0.001 (***).

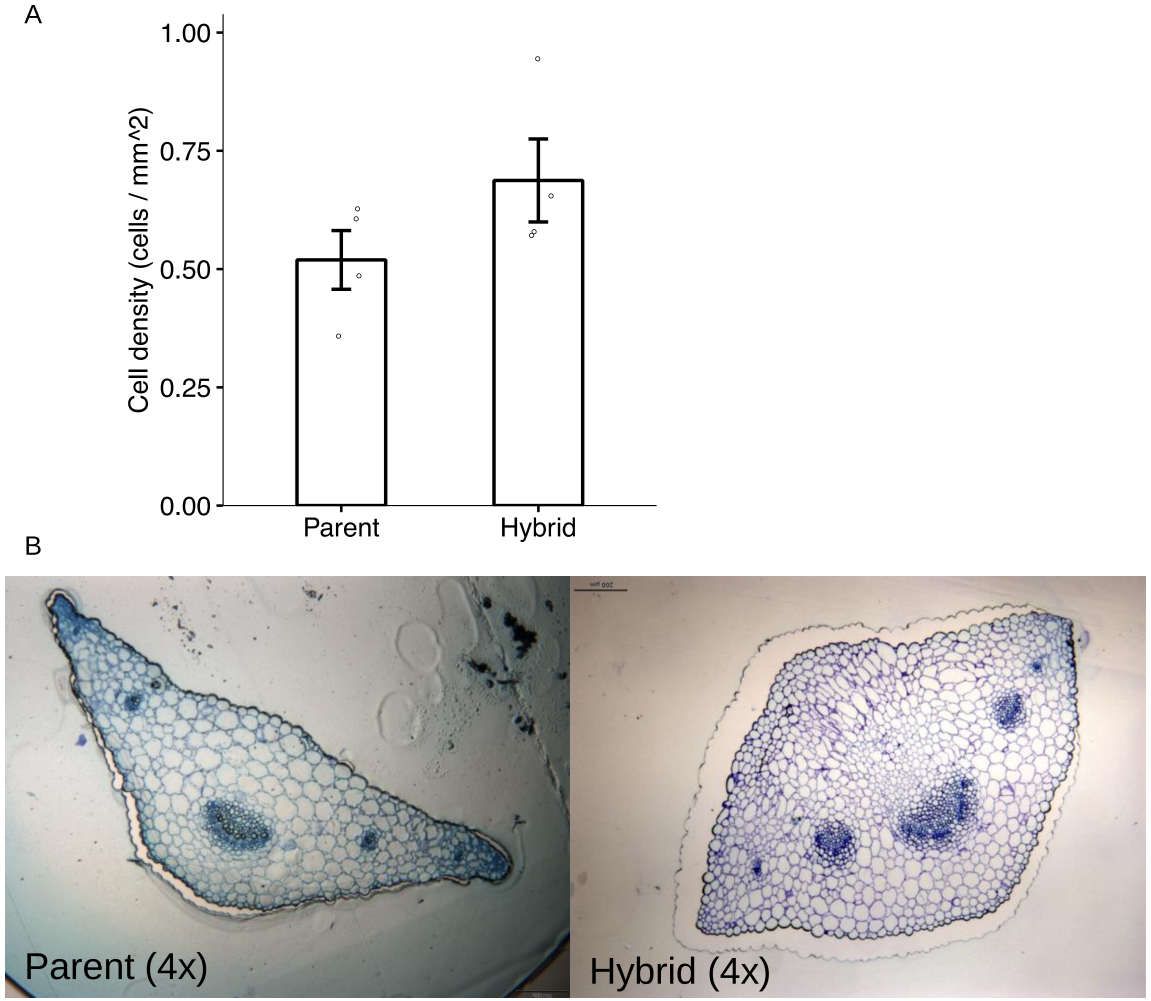

Figure S5. Cell density of transverse section of parent and hybrid petioles. (A) Cell density of transverse sections was measured with ImageJ. N = 4 each genotype, bars represent SE. No statistically significant difference in cell density between parents and hybrids was observed (p-value = 0.09, two-tailed t-test). (B) Example images of sections for parent (left) and hybrid (right).
